# Can We Distinguish Age-Related Frailty from Frailty Related to Diseases? Data from the MAPT Study

**DOI:** 10.1007/s12603-020-1518-x

**Published:** 2020-10-27

**Authors:** Davide Angioni, T. Macaron, C. Takeda, S. Sourdet, M. Cesari, K. Virecoulon Giudici, J. Raffin, W.H. Lu, J. Delrieu, J. Touchon, Y. Rolland, P. De Souto Barreto, B. Vellas, THE MAPT/DSA GROUP

**Affiliations:** 1 Gerontopole of Toulouse, La Grave Hospital, Toulouse University Hospital (CHU Toulouse), Toulouse, France; 2 Gerontopole of Toulouse, Ageing Institut, Toulouse University Hospital (CHU Toulouse), Toulouse, France; 3 Department of Clinical Sciences and Community Health, University of Milan, Milan, Italy; 4 Department of Neurology, University Hospital of Montpellier, Montpellier, France; 5 UPS/Inserm UMR1027, University of Toulouse III, Toulouse, France; 6 Gerontopole of Toulouse, 37 A Jules Guesde, 31000, Toulouse, France

**Keywords:** Frailty origin, frailty related to diseases, age-related frailty, geroscience

## Abstract

**Background:**

No study has tried to distinguish subjects that become frail due to diseases (frailty related to diseases) or in the absence of specific medical events; in this latter case, it is possible that aging process would act as the main frailty driver (age-related frailty).

**Objectives:**

To classify subjects according to the origin of physical frailty: age-related frailty, frailty related to diseases, frailty of uncertain origin, and to compare their clinical characteristics.

**Materials and methods:**

We performed a secondary analysis of the Multidomain Alzheimer Preventive Trial (MAPT), including 195 subjects ≥70 years non-frail at baseline who became frail during a 5-year follow-up (mean age 77.8 years ± 4.7; 70% female). Physical frailty was defined as presenting ≥3 of the 5 Fried criteria: weight loss, exhaustion, weakness, slowness, low physical activity. Clinical files were independently reviewed by two different clinicians using a standardized assessment method in order to classify subjects as: “age-related frailty”, “frailty related to diseases” or “frailty of uncertain origin”. Inconsistencies among the two raters and cases of uncertain frailty were further assessed by two other experienced clinicians.

**Results:**

From the 195 included subjects, 82 (42%) were classified as age-related frailty, 53 (27%) as frailty related to diseases, and 60 (31%) as frailty of uncertain origin. Patients who became frail due to diseases did not differ from the others groups in terms of functional, cognitive, psychological status and age at baseline, however they presented a higher burden of comorbidity as measured by the Cumulative Illness Rating Scale (CIRS) (8.20 ± 2.69; vs 6.22 ± 2.02 frailty of uncertain origin; vs. 3.25 ± 1.65 age-related frailty). Time to incident frailty (23.4 months ± 12.1 vs. 39.2 ± 19.3 months) and time spent in a pre-frailty condition (17.1 ± 11.4 vs 26.6 ± 16.6 months) were shorter in the group of frailty related to diseases compared to age-related frailty. Orthopedic diseases (n=14, 26%) were the most common pathologies leading to frailty related to diseases, followed by cardiovascular diseases (n=9, 17%) and neurological diseases (n = 8, 15%).

**Conclusion:**

People classified as age-related frailty and frailty related to diseases presented different frailty-associated indicators. Future research should target the underlying biological cascades leading to these two frailty classifications, since they could ask for distinct strategies of prevention and management.

## Glossary

**Chronic disease potentially leading to physical frailty**: chronic disease capable of explaining the onset of ≥ 3 Fried's criteria.
**Age-related frailty**: The occurrence of physical frailty not related to the comorbidities known at baseline or to the clinical events occurring during the follow-up period, but rather occurring with advancing age.
**Frailty related to diseases**: The occurrence of physical frailty related to one or more intervening medical events occurring during followup period.
**Frailty of uncertain origin**: Uncertain relationship between the occurrence of physical frailty and the medical history (comorbidities and intervening medical events occurring during the follow-up period).
**Intervening medical event**: one of the following events: 1) acute illness leading to medical consultation and/or emergency visit and/or hospitalization; 2) clinically significant worsening of a chronic disease known at baseline; 3) diagnosis of a new chronic disease.
**Significant worsening of a chronic disease known at baseline**: active chronic disease already known at baseline that worsens during the follow-up period and becomes capable of explaining the onset of ≥3 Fried's criteria (e.g. worsening of cardiac function in a context ofischemic heart disease leading to heart failure).
**Major impact event**: an event capable of explaining the onset of ≥3 Fried's criteria, with a likely clinical and temporal relationship between the event and the occurrence of physical frailty criteria (e.g. metastatic cancer followed by weight loss, low level of physical activity and fatigue).
**Uncertain impact**: an event capable of explaining the occurrence of physical frailty but with a doubtful clinical or temporal relationship (e.g. subject sedentary at baseline, with new onset of depression followed by fatigue and weakness).
**Minor impact**: an event that could not explain the occurrence of physical frailty.

## Background

Evolving chronic diseases have been suggested to contribute to the development of physical frailty (1)(2)(3). Several cross-sectional and longitudinal studies have shown associations between physical frailty and chronic diseases, such as cardiovascular disease (4)(5), neurological diseases (6) (7), systemic diseases (8), chronic infections (9) and cancer (10). Acute illnesses and injuries leading to hospitalization have been associated with the transition from non-frail to frail status (11)(12)(13)(14). Although both chronic diseases and acute illnesses have been related to physical frailty, in clinical practice it is common to observe frail subjects (presenting ≥3 of the 5 Fried criteria) without evolving diseases or any context of an acute illness. In these cases, we can hypothesize that the aging process could play a major role on the onset of physical frailty. Indeed, the aging process is associated with a progressive homeostatic and homeodynamic dysregulation responsible for the loss of the resilience capacity, increasing individual's susceptibility to develop or worsen a frailty status (15). Therefore, we hypothesized that several paths may lead to physical frailty, being a disease-related and an aging-related two of the most common causes of physical frailty.

To the best of our knowledge, no study has tried to distinguish subjects who become frail probably due to diseases (frailty related to diseases) from those who develop frailty in the absence of specific medical events; in this latter case, it is possible the aging process would represent the main frailty driver (age-related frailty). Age-related frailty and frailty related to diseases could have different underlying biological mechanisms, which could lead to different clinical characteristics and trajectories over time, asking for distinct strategies of prevention and management. The objective of this preliminary study was to classify community-dwelling older adults who developed physical frailty over a 5-year follow-up into three groups (ie, “frailty related to diseases”, “age-related frailty” and “frailty of uncertain origin”), and to compare their clinical characteristics.

## Methods

### The MAPT study

The present study used data of the Multidomain Alzheimer Preventive Trial (MAPT). The MAPT Study (registration: NCT00672685) was a randomized controlled trial (RCT) aiming to assess the effects of multidomain interventions (nutritional and physical activity counselling, and cognitive training), omega-3 supplementation, or their combination on cognitive function over 3 years. The trial found no effect of these interventions on a composite cognitive score. Participants were additionally followed for 2 years. MAPT methods and procedures have been previously described (16)(17)(18). The trial respected the Declaration of Helsinki and was approved by the ethics committee in Toulouse (CPP SOOM II). Written consent was obtained.

### Participants and follow-up

MAPT participants were community-dwelling subjects aged 70 years old or more, without dementia and who met at least one of the following criteria: limitation in executing ≥1 Instrumental Activity of Daily Living, spontaneous memory complaints, slow gait speed (≤ 0.8 m/s). In this analysis, we included all MAPT participants who were robust or pre-frail at baseline and who became frail during the 5-year follow-up.

Participants underwent clinical, functional, psychological, cognitive and frailty assessments at baseline and at 6, 12, 24, 36, 48 and 60 months. Visits consisted of a consultation with a physician that included a physical examination, and a blood sample analysis. At baseline evaluation, past and current comorbidities were recorded. During the follow-up, all intervening medical events (hospitalizations, emergency visits, acute illnesses, progression of an existing disease, onset of a new chronic disease) as well as information on the treatments (starting treatment or cessation, dose adjustments) were recorded. Other medical elements (e.g., general practitioner or specialty consultations, laboratory or radiological exams) and information on death and any other reason for premature study discontinuation were also recorded.

### Physical Frailty assessment

Physical Frailty was determined according to the following five criteria of Fried frailty phenotype (19):

• Unintentional weight loss (>4.5 kg) in the past year;
• Fatigue measured by two questions from the Center for Epidemiologic Studies Depression Scale (CES-D) depression scale
• Low hand grip strength based on the best of three measurements with preferred hand;
• Slow walking speed based on the best of two measurements over 4 meters;
• Low level of physical activity expressed in weekly energy expenditure relating to time spent doing physical activities.

Baseline frailty status was categorized as follows: frail (meeting ≥3 frailty criteria), pre-frail (meeting one or two criteria), robust (no criterion). People with physical frailty at baseline, without frailty assessment at the follow-up, and those not developing physical frailty were not included in the present study. In order to better characterize the trajectories of frailty during follow-up, time to incident frailty was calculated as the time (months) between the first visit and the occurrence of physical frailty. Time of pre-frailty (months) was defined as the period, during the follow-up, in which the subjects had a pre-frail status.

### Clinical files' review

Given the lack of previous research exploring the potentially different origins of physical frailty, a working-group, composed of six experienced clinicians (BV)(YR)(SS)(CT)(DA)(TM) in the field of frailty, developed a standardized method for data source use and a decisional flow chart to classify subjects in one of three groups: age-related frailty, frailty related to disease, and frailty of uncertain origin. Detailed definition of terms are presented in the Glossary. Age-related frailty and frailty related to diseases are not consensual terms, but based on our hypothesis about the origin of physical frailty. We used the term “age-related frailty” as for the cognitive decline it was referred as “age-related cognitive decline” (20). We used the term “frailty of uncertain origin” because for a proportion of subjects it was not possible to conclude.

### Standardized method for data source use

For each subject, demographic data (age, sex, place of living) and clinical data were considered at baseline, during the follow-up period, and up to 1 year following the date of frailty occurrence. This 1-year period was decided because the discovery of physical frailty frequently led to additional investigations allowing the diagnosis of underlying causes. Considering clinical data, we focused on: past and current comorbidities at baseline; all available clinical assessments: cognitive status assessed by the Mini-Mental State Examination (MMSE), functional status assessed by the Short Physical Performance Battery (SPPB), nutritional status assessed by the Body Mass Index (BMI), psychological status assessed by the Geriatric Depression Scale (GDS) and physical frailty assessed by Fried criteria; the intervening medical events (e.g., fall followed by fracture, hospitalization due to urinary tract infection, diagnostic of cancer); drug treatments and all medical records (e.g., general practitioner specialty or emergency consultations, laboratory or radiological exams) available in the clinical file of the subject.

### Decisional flow-chat to classify subjects

**Figure 1:**
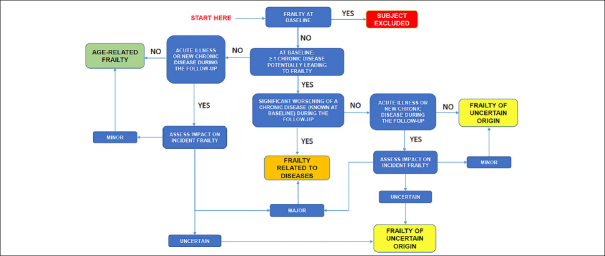
Decisional flow-chat

Figure 1 illustrates the flow-chart used to classify subjects. Based on clinical experience, we defined a chronic disease potentially leading to physical frailty as a disease capable of explaining the onset of ≥ 3 Fried's criteria (eg. symptomatic peripheral artery occlusive disease, uncontrolled diabetes, active connective tissue disease). We considered an intervening medical event during the follow up period as:

• an acute illness leading to medical consultation and/or emergency department admission and/or hospitalization (e.g., lower limb fractures, strokes);
• a significant worsening of a chronic disease known at baseline (glossary), according to the clinical appreciation of the physician (e.g., a person with stable ischemic heart disease at baseline who presented several episodes of acute pulmonary edema due to the deterioration of cardiac function);
• the diagnosis of a new chronic disease (e.g., cancer).

All self-reported intervening medical events and/or confirmed by medical records (registered in the clinical file of the subject) were considered. Each event was classified as having major, minor or uncertain impact (glossary). For each event, we analyzed the need for an emergency visit and/or hospitalization; the length of the hospitalization; the admission to Intensive Care Unit; the complications eventually associated with hospitalization (e.g. functional decline, weight loss, depression) and the temporal relationship with the occurrence of physical frailty.

### Classification procedure

The classification process was organized in two phases. In the first phase, two investigators (geriatricians) (DA)(TM) independently reviewed the clinical files of all the subjects included in the study in order to classify them into three groups: “frailty related to diseases”, “age-related frailty” and “frailty of uncertain origin”. When the two investigators were faced with a disagreement, or if files were classified as “frailty of uncertain origin” by both investigators, they were considered as “unclassified” during this first phase. In the second phase, in order to improve the classification, “unclassified files” were reviewed by two more experienced geriatricians (CT)(BV).

### Statistical analysis

Descriptive statistics for the three groups (ie, frailty related to diseases, age-related frailty, or frailty of uncertain origin) were presented as mean (standard deviation — SD) or absolute values (%), as appropriate. Demographic (age, sex, place of living) and clinical characteristics (CIRS-G score (21), BMI, SPPB and its items, GDS, MMSE) at baseline, time to incident frailty, time of pre-frailty and the Fried criteria were compared across groups using chi-square test for qualitative variables and One-way ANOVA test for quantitative variables. Significant chi-square and ANOVA tests were followed by post-hoc tests for pairwise comparisons. Post-hoc tests were then adjusted for multiple comparisons using the Benjamini-Hochberg procedure (22). Two-sided p-values < 0.05 were considered as statistically significant. All analyses were performed using SPSS statistics version 23.

## Results

Among the 1679 subjects enrolled in MAPT Study, 91 were excluded because of missing data on frailty and 51 because they were frail at baseline. Among the 1537 non frail patients at baseline, 195 (12.6 %) became frail after a mean period of 32.5 (±18.5) months. In the first phase of the classification process, 127 subjects (65%) were classified: 46 subjects (36%) as “frail related to diseases”, and 81 (64%) as “age-related frailty”. Sixty-eight (35%) were unclassified and reconsidered in the second phase of the classification process. During the second phase, 7 (10%) unclassified participants were classified as “frailty related to diseases”, 1 (1%) as “age-related frailty” and 60 (79%) as “frailty of uncertain origin”. At the end of the process of classification, 82 subjects (42%) were classified as “age-related frailty”, 53 (27%) as “frailty related to diseases” and 60 (31%) as “frailty of uncertain origin”.

**Figure 2:**
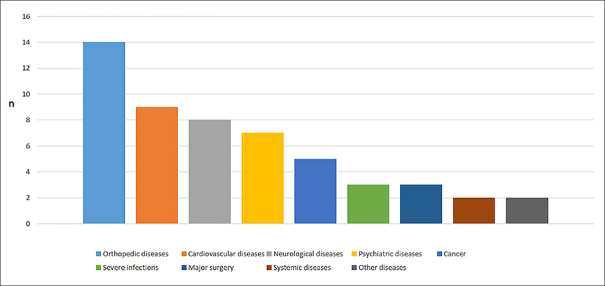
Pathologies responsible of frailty in the group frailty related to diseases

Clinical and demographic characteristics of the population according to the origin of physical frailty are presented in Table 1. Mean age was 77.8 years ± 4.7, 70% were female, and one fourth of patients were robust at baseline. Subjects included in this study presented a high cognitive and a satisfying functional status at baseline. Comparing the 3 groups according to the origin of physical frailty, we did not find any significant differences in terms of MMSE, SPPB and its items, BMI, GDS, but participants with frailty related to diseases presented a higher burden of comorbidity as measured by CIRS-G (8.20 ± 2.69 vs. 6.22 ± 2.02 frailty of uncertain origin; vs. 3.25 ± 1.65 age related frailty, p < 0.0001). The time to incident frailty (23.4 ± 12.1 vs. 39.2 ± 19.3 months, adjusted-p < 0.0001) and the time spent on a pre-frail status (17.1 ± 11.4 vs 26.6 ± 16.6 months, adjusted-p < 0.0001) were shorter in the group “frailty related to diseases” compared to the group “age-related frailty”. The criterion weight loss was more common in the group “frailty related to disease” compared to the other groups (43%; vs. 26% age-related frailty, vs 20% frailty of uncertain origin, p = 0.020). Considering the group with frailty related to disease, the most common disease categories leading to frailty were notably orthopedic, cardiovascular and neurological conditions (Figure 2).

**Table 1:** Clinical and demographic characteristics

	**Total n = 195**	**Frailty related to diseases n = 53**	**Age-related Frailty n = 82**	**Frailty of uncertain origin n = 60**	**Unadjusted p values**	**Post hoc adjusted comparaison**
*CHARACTERISTICS AT BASELINE*						
Age (years)	77.85 ± 4.7	78.7 ± 5.1	77.7 ± 4.7	77.2 ± 4.3	0.232	
Sex (female)	59(70%)	19(65%)	25(70%)	15(75%)	0.926	
MAPT GROUP					0.557	
Multidomain intervention and n-3 PUFA supplementation	41(21%)	10(18%)	21(25%)	10(16%)		
n-3 PUFA supplementation	53(27%)	17(32%)	23(28%)	13(21%)		
Multidomain intervention	45(23%)	13(24%)	17(20%)	15(25%)		
Placebo	56(28%)	13(24%)	21(25%)	22(36%)		
CIRS score	5.51 ± 2.93	8.20 ± 2.69	3.25 ± 1.65	6.22 ± 2.02	<0.0001	* ** ***
BMI	25.98 ± 3.94	25.64 ± 3.83	26.45 ± 4.12	25.65 ± 3.79	0.374	
Fried Frailty status (Robust subjects)	50(25%)	12(22%)	29(35%)	9(15%)	0.019	***
MMSE score	27.85 ± 1.62	27.64 ± 1.61	28.01 ± 1.58	27.80 ± 1.77	0.432	
ADL	6	6	6	6	-	
GDS score	4.22 ± 2.89	3.94 ± 3.16	4.09 ± 2.55	4.65 ± 3.06	0.375	
SPPB score	9.60 ± 1.89	9.38 ± 1.87	9.88 ± 1.81	9.41 ± 2.00	0.218	
Walking speed (m/s)	1.13 ± 0.28	1.10 ± 0.26	1.13 ± 0.27	1.17 ± 0.31	0.440	
Chair stand time (seconds)	14.17 ± 5.34	14.86 ± 6.78	13.25 ± 4.13	14.76 ± 5.21	0.147	
Balance test (from SPPB)	3.35 ± 0.92	3.20 ± 1.04	3.47 ± 0.78	3.31 ± 0.98	0.605	
*EVOLUTION*						
Time of pre-frailty (months)	22.58 ± 15.14	17.09 ± 11.37	26.56 ± 16.68	22.00 ± 15.37	0.020	
Time to incident frailty (months)	32.46 + 18.50	23.43 ± 12.13	39.22 ± 19.30	31.20 ± 18.61	<0.0001	
Weight loss	57 (29%)	23 (43%)	22 (26%)	12 (20%)	0.020	* **
Exhaustion	158 (81 %)	44 (83%)	65 (79%)	49 (81%)	0.853	
Weakness	163 (83%)	41 (77%)	73 (89%)	49 (81%)	0.180	
Slowness	98 (50%)	32 (60%)	36 (43%)	30 (50%)	0.174	
Low Physical activity	142 (72%)	38 (71%)	60 (73%)	44 (73%)	0.977	


Numbers represent mean and standard deviation or frequency and percentages as appropriate; ADL = Activities of daily living; BMI = Body mass index; CIRS = Cumulative Illness Rating Scale; GDS = Geriatric Depression Scale; MMSE = Mini Mental State Examination; n-3 PUFA = omega 3 polyunsaturated fatty acid; SPPB = Short Physical Performance Battery; *Frailty related to disease vs. Age-related frailty: adjusted-p < 0.05; **Frailty related to disease vs. Frailty of uncertain origin: adjusted-p < 0.05; ***Age-related frailty vs. frailty of uncertain origin: adjusted-p < 0.05

## Discussion

We performed the first study aimed to classify community-dwelling older subjects who presented an incident frailty over a 5-year follow-up, according to the origin of physical frailty, in three groups: “frailty related to diseases”, “age-related frailty” and “frailty of uncertain origin”. Age-related frailty was the most prevalent classification of frailty followed by frailty of uncertain origin and frailty due to diseases. These three groups did not differ for clinical characteristics at baseline (except for comorbidity burden), but presented different trajectories leading to frailty: the time to incident frailty and the time spent in a pre-frailty condition were longer in the group with age-related frailty compared to frailty related to diseases.

We found that 42% of subjects became frail in the absence of intervening medical events potentially leading to frailty. The fact that age-related frailty was the most prevalent classification of frailty could have been due to the clinical characteristics of our healthy population. Indeed we included participants with few comorbidities at baseline, and relatively high cognitive and physical status. Considering the group frailty related to diseases, the first group of pathologies responsible for frailty were orthopedic diseases. Among them, 50% of cases were a fracture of the lower limb. As expected, this was a major cause of loss of autonomy and transition to frailty status. About one third of cases in our sample were classified as “frailty of uncertain origin”. This shows the complex relationship between diseases, aging and frailty (1)(23). Indeed, frailty is a complex syndrome, which could result from the interaction of clinical, physical, psychological, cognitive and social factors (15)(24) (25). It is possible that the proportion of uncertain cases could have been overestimated due to the design of our study; as clinical files were analyzed retrospectively. In some cases, we were confronted to the lack of the information needed for the classification process, and it was not possible to recover the data.

Participants with age-related frailty and frailty related to diseases did not differ for clinical characteristics at baseline (except for comorbidity burden) but they presented different trajectories leading to frailty. People in the group frailty related to diseases became frail faster, after a reduced time of pre-frailty, presenting a vertical (or rapid progression) trajectory. In contrast, for those with age-related frailty, the time to frailty was slower and the time of pre-frailty was longer, illustrating a more horizontal (or slower progression) trajectory, leaving potentially more time for integrated care interventions. Considering the different frailty criteria, we found that the weight loss criterion was more common in the group with frailty related to diseases compared to others groups. This is not surprising, given that unintentional weight loss is a common sign in several diseases in older adults (26)(27). Our findings suggest that, if the incident frailty is associated with weight loss, further additional investigations are needed for the diagnosis of underlying causes of both conditions.

Although we did not find major differences from a clinical point of view, it is possible that the underlying biological mechanisms involved in the onset of frailty related to diseases are different from those involved in age-related frailty. Several biological mechanisms have been suggested for the pathogenesis of frailty, such as inflammation, endocrinological and genetic changes (28). However, to the best of our knowledge, in the context of frailty there are no biomarkers able to differentiate the changes related to aging and those related to diseases (28)(29)(30). Moreover, geroscience could help define the concept of age-related frailty, and to establish the main processes leading to frailty in the absence of evolving chronic diseases and intervening medical events. In this sense, further longitudinal translational studies, including the use of animal models of frailty (31), could help figuring out if frailty could happen mainly due to the intrinsic biological mechanism of aging.

This study has several strengths that extend the literature on the topic: it is a preliminary study aimed to classify subjects according to the origin of physical frailty, and it proposes a methodology to perform this classification. Nevertheless, there are several limitations that should be acknowledged. Given the relatively small sample of the study, we decided not to exclude subjects that underwent multidomain interventions, so we cannot exclude their potential impact on the onset of physical frailty. Our study is a secondary analysis of the MAPT study, thus, it was not specifically designed to test our hypothesis about the origin of physical frailty. Lastly, the lack of clinical data due to retrospective analysis of the clinical files could have impacted the classification process, leading to an overestimation of frailty with uncertain origin. In order to properly evaluate main causes of incident frailty, it would be important to apply the classification procedures prospectively. For this reason, we are implementing the identification and description of age-related frailty and frailty related to diseases in the on-going translational cohort of the INSPIRE Project (32, 33, 34, 35).

## Conclusion

In this preliminary study, we distinguished community-dwelling older adults presenting an incident frailty during a 5-year follow-up according to its origin: “age-related frailty”, “frailty related to diseases” and “frailty of uncertain origin”. Participants with age-related frailty were the most prevalent, and presented different frailty-associated indicators compared to participants with frailty related to diseases: a slower trajectory leading to frailty with a longer phase of pre-frailty. Future research should target the underlying biological cascades leading to these two frailty classifications, since they could ask for distinct strategies of prevention and management.

## MaPT/Dsa group refers to MAPT Study Group

*Principal investigator:* Bruno Vellas (Toulouse); *Coordination:* Sophie Guyonnet. *Project leader:* Isabelle Carrié. *CRA:* Lauréane Brigitte. *Investigators:* Catherine Faisant, Françoise Lala, Julien Delrieu, Hélène Villars; *Psychologists:* Emeline Combrouze, Carole Badufle, Audrey Zueras. *Methodology, Statistical analysis and data management:* Sandrine Andrieu, Christelle Cantet, Christophe Morin. *Multidomain group:* Gabor Abellan Van Kan, Charlotte Dupuy, Yves Rolland (physical and nutritional components), Céline Caillaud, Pierre-Jean Ousset (cognitive component), Françoise Lala (preventive consultation) (Toulouse). The cognitive component was designed in collaboration with Sherry Willis from the University of Seattle, and Sylvie Belleville, Brigitte Gilbert and Francine Fontaine from the University of Montreal. *Co-Investigators in associated centres:* Jean-François Dartigues, Isabelle Marcet, Fleur Delva, Alexandra Foubert, Sandrine Cerda (Bordeaux); Marie-Noëlle-Cuffi, Corinne Costes (Castres); Olivier Rouaud, Patrick Manckoundia, Valérie Quipourt, Sophie Marilier, Evelyne Franon (Dijon); Lawrence Bories, Marie-Laure Pader, Marie-France Basset, Bruno Lapoujade, Valérie Faure, Michael Li Yung Tong, Christine Malick-Loiseau, Evelyne Caz ab an-Campistron (Foix); Françoise Desclaux, Colette Blatge (Lavaur); Thierry Dantoine, Cécile Laubarie-Mouret, Isabelle Saulnier, Jean-Pierre Clément, Marie-Agnès Picat, Laurence Bernard-Bourzeix, Stéphanie Willebois, Iléana Désormais, Noëlle Cardinaud (Limoges); Marc Bonnefoy, Pierre Livet, Pascale Rebaudet, Claire Gédéon, Catherine Burdet, Flavien Terracol (Lyon), Alain Pesce, Stéphanie Roth, Sylvie Chaillou, Sandrine Louchart (Monaco); Kristelle Sudres, Nicolas Lebrun, Nadège Barro-Belaygues (Montauban); Jacques Touchon, Karim Bennys, Audrey Gabelle, Aurélia Romano, Lynda Touati, Cécilia Marelli, Cécile Pays (Montpellier); Philippe Robert, Franck Le Duff, Claire Gervais, Sébastien Gonfrier (Nice); Yannick Gasnier and Serge Bordes, Danièle Begorre, Christian Carpuat, Khaled Khales, Jean-François Lefebvre, Samira Misbah El Idrissi, Pierre Skolil, Jean-Pierre Salles (Tarbes). *MRI group:* Carole Dufouil (Bordeaux), Stéphane Lehéricy, Marie Chupin, Jean-François Mangin, Ali Bouhayia (Paris); Michèle Allard (Bordeaux); Frédéric Ricolfi (Dijon); Dominique Dubois (Foix); Marie Paule Bonceour Martel (Limoges); François Cotton (Lyon); Alain Bonafé (Montpellier); Stéphane Chanalet (Nice); Françoise Hugon (Tarbes); Fabrice Bonneville, Christophe Cognard, François Chollet (Toulouse). *PET scans group:* Pierre Payoux, Thierry Voisin, Julien Delrieu, Sophie Peiffer, Anne Hitzel, (Toulouse); Michèle Allard (Bordeaux); Michel Zanca (Montpellier); Jacques Monteil (Limoges); Jacques Darcourt (Nice). *Medico-economics group:* Laurent Molinier, Hélène Derumeaux, Nadège Costa (Toulouse). *Biological sample collection:* Bertrand Perret, Claire Vinel, Sylvie Caspar-Bauguil (Toulouse). *Safety management:* Pascale Olivier-Abbas. *DSA Group:* Sandrine Andrieu, Christelle Cantet, Nicola Coley.
